# West Texas Medical Association

**Published:** 1902-10

**Authors:** W. B. Russ

**Affiliations:** Secretary


### West Texas Medical Association.

The September meeting of the West Texas Medical Association was held on Thursday night, the 25th.

The meeting was called to order by President Luter.

Roll call showed the following members to be present: Col. P. T. Cleary, U. S. A.; Drs. P. Baldesarilli, James Bartlett, H. D. Barnitz, D. Berry, S. Burg, H. J. Chapman, A. D. Dupuy, A. Garwood, F. M. Hicks, T. T. Jackson, J. K. Kenney, C. E. R. King, B. F. Kingsley, W. E. Luter, Charles F. Mason, F. Paschal, R. Robinson, W. B. Russ, L. L. Shropshire, Clarence Warfield, G. G. Watts, W. M. Wolf, E. Cross, A. P. D. Cleary, J. A. Burke, T. S. Bratton, B. Ferguson and T. P. Lloyd.

Dr. L. L. Shropshire, chairman of the committee appointed some weeks ago to wait upon the school board for the purpose of requesting that body, in the interest of public health, to increase the time for the noon recess in the public schools, reported that the resolutions upon the subject as adopted by the medical society had been presented to the school board, but that the demands of the society had not been given consideration. He suggested that the matter should be brought before the State Medical Society for the purpose of demonstrating to the public that no physician in possession of his senses will endorse a one-half hour noon recess for young children. All present agreed with Dr. Shropshire.

Dr. Kingsley, for the Committee on Arrangements for the twenty-seventh annual meeting, reported that the date had been set for the morning, afternoon and evening of Thursday, October 30th, and that the Y. M. C. A. hall had been secured to accommodate the large number of members and visitors expected. The evening session will be followed by a banquet, at which Dr. C. E. R. King will serve as toastmaster.

Dr. J. W. Kenney reported a case of peripheral neuritis following an injury to the sciatic nerve. Drs. Jackson, Paschal, Cross and Cleary discussed this paper.

On motion by Col. Cleary, surgeon, U. S. A., seconded by Drs. Cross, Wolf and Burg, the following resolution was unanimously adopted:

"*Resolved,* That having heard Dr. Berry's excellent paper and the report of the investigating committee appointed to examine Vicento Saucedo, we are convinced that Saucedo was an epileptic and represented a very low order of intelligence."

W. B. Russ, M. D., Secretary.

[Saucedo was hanged very lately for rape of his ten year old step-daughter, epilepsy or no epilepsy.—Ed.]

---

